# CiRS‐7 targeting miR‐7 modulates the progression of non‐small cell lung cancer in a manner dependent on NF‐κB signalling

**DOI:** 10.1111/jcmm.13587

**Published:** 2018-03-13

**Authors:** Chongyu Su, Yi Han, Hongtao Zhang, Yunsong Li, Ling Yi, Xiaojue Wang, Shijie Zhou, Daping Yu, Xiaoyun Song, Ning Xiao, Xiaoqing Cao, Zhidong Liu

**Affiliations:** ^1^ Department of Thoracic Surgery Beijing Chest Hospital Capital Medical University Beijing China; ^2^ Department of Central Laboratory Beijing Chest Hospital Capital Medical University and Beijing Tuberculosis and Thoracic Tumor Research Institute Beijing China

**Keywords:** cell activity, cell apoptosis, cell invasion, cell migration, CiRS‐7, miR‐7, NF‐kB, non‐small cell lung cancer

## Abstract

The purpose of this study was to figure out the effect of ciRS‐7/miR‐7/NF‐κB axis on the development of non‐small cell lung cancer (NSCLC). In response, the expressions of ciRS‐7, miR‐7 and NF‐κB subunit (ie RELA) within NSCLC tissues and cell lines were determined with real‐time polymerase chain reaction (RT‐PCR) and Western blot. Moreover, the NSCLC cells were transfected with pcDNA3‐ciRS‐7‐ir, pcDNA3‐ciRS‐7, miR‐NC and miR‐7 mimic. Furthermore, the targeted relationships between ciRS‐7 and miR‐7, as well as between miR‐7 and RELA, were confirmed by luciferase reporter assay. The proliferation, migration and apoptosis of NSCLC cells were, successively, measured using CCK‐8 assay, wound‐healing assay and flow cytometry test. Consequently, ciRS‐7, miR‐7, histopathological grade, lymph node metastasis and histopathological stage could independently predict the prognosis of patients with NSCLC (all *P* < .05). Moreover, remarkably up‐regulated ciRS‐7 and RELA expressions, as along with down‐regulated miR‐7 expressions, were found within NSCLC tissues and cells in comparison with normal ones (*P* < .05). Besides, overexpressed ciRS‐7 and underexpressed miR‐7 were correlated with increased proliferation, migration and invasion, yet reduced apoptosis rate of NSCLC cells (*P* < .05). More than that, ciRS‐7 specifically targeted miR‐7 to reduce its expressions (*P* < .05). Ultimately, the NSCLC cells within miR‐7 + RELA group were observed with superior proliferative, migratory and invasive capabilities than those within miR‐7 group (*P* < .05), and RELA expression was also significantly modified by both ciRS‐7 and miR‐7 (*P* < .05). In conclusion, the ciRS‐7/miR‐7/NF‐kB axis could exert pronounced impacts on the proliferation, migration, invasion and apoptosis of NSCLC cells.

## 1 INTRODUCTION

Lung cancer is a common human malignancy with leading prevalence and mortality worldwide, and non‐small cell lung cancer (NSCLC) accounted for as high as 80%‐85% of all lung cancer cases.^1^, ^2^ Furthermore, the treatment efficacy and 5‐year survival rate for NSCLC patients were usually undesirable, as NSCLC cases were mostly diagnosed when they have developed into the advanced stage.^3^ Thus, it was urgently demanded to seek for a screening technique for early‐stage NSCLC that was potentially non‐invasive, little traumatized and highly specific.

Notably, exosomes that were shaped as bilayer vesicles were viewed within tumour cells, and their formation was based on a series of regulatory processes, including endocytosis, fusion and efflux.^4^, ^5^, ^6^ The exosomes abounded in RNA, protein, microRNA and DNA segments, which participated in regulating the biological behaviour of recipient cells.^7^, ^8^ Furthermore, the non‐coding regions of DNA segments (eg miRNA, lncRNA and circRNA) accounted for as high as 98% of human genomes, and they were involved with altering the epigenetic inheritance of neoplasms.^9^, ^10^, ^11^ It was thus implied that the non‐coding regions, especially circRNAs, within exosomes might be advantageous in precisely discriminating tumours at early stage. For instance, plenty of intact and stable circRNAs were discovered within human serum exosomes, and therein the circ‐KLHDC10 expression showed evident distinctions between colorectal cancer patients and healthy individuals.^12^ In addition, ciRS‐7 (also termed as Cdr1as) not merely exhibited a higher expression within hepatocellular carcinoma (HCC) tissues than within para‐carcinoma tissues, but also was significantly correlated with hepatic microvascular invasion (MVI) among patients with NSCLC.^13^ Therefore, ciRS‐7 was speculated as potential biomarker for diagnosing HCC or NSCLC. Interestingly, ciRS‐7 expressions displayed an inverse correlation with miR‐7 expressions within tumour tissues, and ciRS‐7 has also been verified to directly target miR‐7 within tumour cells.^13^, ^14^, ^15^ Nonetheless, the mechanism underlying the correlation between ciRS‐7 and miR‐7 within NSCLC cells was still far from clear. Furthermore, miR‐7 also participated in the pathogenesis that initiated the development of assorted disorders through acting on the downstream pathways. To be specific, miR‐7 could affect proliferation and migration of NSCLC cells by modulation of ERK/MAPK signalling or mediation of TLR9 signalling.^16^, ^17^ The miR‐7/NF‐κB signalling axis also mattered for its modulating the metastatic conditions of gastric cancer cells, so this axis might also be responsible for the aetiology of additional cancers, such as NSCLC.^18^, ^19^, ^20^ In consequence, there could exist a correlation among ciRS‐7, miR‐7, NF‐κB signalling and NSCLC onset.

Above all, as few studies systematically incorporated ciRS‐7, miR‐7 and NF‐κB regarding their functions for NSCLC onset, this study was aimed to unravel whether ciRS‐7 secreted by exosomes would alter NSCLC development via regulation of downstream miR‐7 and NF‐κB signalling.

## 2 MATERIALS AND METHODS

### 2.1 Collection of NSCLC tissues

From June 2013 to December 2016, 128 pairs of NSCLC tissues and para‐carcinoma tissues were collected from Beijing Chest Hospital affiliated to Capital Medical University. The fresh specimens were cryopreserved at −80°C within 30 minutes after exairesis. All cases were pathologically confirmed by >2 senior professionals, and none of them had received chemotherapy or radiation before surgery. The histopathological grading of NSCLC was evaluated in light of World Health Organization (WHO),^21^ and NSCLC was appraised based on tumour‐node‐metastasis (TNM) classification system of International Union Against Cancer.^22^, ^23^ Each recruited subject has signed informed consents, and this protocol has obtained approvals of Beijing Chest Hospital affiliated to Capital Medical University and the ethics committee of Beijing Chest Hospital affiliated to Capital Medical University.

### 2.2 Cell culture

Human NSCLC cell lines (ie A549, H1299, H1355 and H460) and the normal human embryonic lung fibroblast cell line (ie MRC5) were purchased from Shanghai Institute of Biochemistry and Cell biology, Chinese Academy of Sciences. All the cell strains were inoculated within cell medium that contained 10% bovine foetal serum, 2 μmol/L glutamine, 100 IU/mL penicillin and 100 μg/mL streptomycin sulphate. Subsequently, the cells were managed to grow in 5% CO_2_ and saturated humidity at 37°C.

### 2.3 Real‐time fluorescence quantification polymerase chain reaction (RT‐qPCR)

Total RNA was extracted by consulting the operational manual of routine TR1201 reagent (Invitrogen Corporation, USA), and the extracted RNAs were preserved at −80°C. Then, the purity and concentration of RNA were measured by applying spectrophotometry. Moreover, the expressions of miR‐7 and ciRS‐7 were measured through qRT‐PCR (Invitrogen Corporation), and their primers were designed and composed by Sangon Biotech (Shanghai) Corporation, China (Table 1). GAPDH was set as the reference gene of ciRS‐7, and U6 was set as the reference gene of miR‐7. The PCR reaction conditions for ciRS‐7 were specifically displayed as: (i) 95°C for 5 seconds; (ii) 40 cycles of 95°C for 5 seconds and 60°C for 35 seconds; (iii) 95°C for 15 seconds, 60°C for 60 seconds and 95°C for 15 seconds; and (iv) 95°C for 15 minutes. For another, the PCR reaction conditions for miR‐7 were enlisted as: (i) 95°C for 10 minutes; (ii) 40 cycles of 95°C for 15 seconds and 60°C for 30 seconds; (iii) 95°C for 15 seconds, 60°C for 60 seconds and 95°C for 15 seconds; and (iv) 95°C for 15 minutes. The 2^−▵▵Ct^ method was used to calculate relative quantification (RQ) values, and each experiment was repeated for at least 3 times.

**Table 1:** The primers for ciRS‐7, miR‐7, U6, NFκB and GAPDH investigated in this study

RNAs	Primer sequence
ciRS‐7	
Forward	5′‐ACGTCTCCAGTGTGCTGA‐3′
Reverse	5′‐CTTGACACAGGTGCCATC‐3′
miR‐7	
Forward	5′‐CTAGCTAGCTAGAGCACCAATAGGGAAGGG‐3′
Reverse	5′‐GAAGATCTTCGAGTCTGCCGATGGGTGT‐3′
U6	
Forward	5′‐CTCGCTTCGGCAGCACA‐3′
Reverse	5′‐AACGCTTCACGAATTTGCGT‐3′
NFκB	
Forward	5′‐CCCCACGAGCTTGTAGGAAAG‐3′
Reverse	5′‐CCAGGTTCTGGAAACTGTGGAT‐3′
GAPDH	
Forward	5′‐TGCACCACCAACTGCTTAGC‐3′
Reverse	5′‐GCATGGACTGTGGTCATGAG‐3′

### 2.4 Cell transfection

The pcDNA3‐ciRS‐7‐ir and pcDNA3‐ciRS‐7 were granted by T. Hansen (Aarhus university, Denmark).^24^ With aid of the transfection reagent Lipofectamine TM 2000, NSCLC cells were, respectively, transected with pcDNA3‐ciRS‐7‐ir, pcDNA3‐ciRS‐7, miR‐NC and miR‐7 mimic, and then they were collected about 24‐48 hours after transfection.

### 2.5 Trypan blue staining

The cells at the exponential phase were digested with pancreatins, and they were processed into cell suspension after low‐speed centrifugation. Subsequently, after being mixed with the trypan blue saline solution (percentage: 0.4%), they were dropped upon the blood counting chamber, and cell counting was completed within 3 minutes. Finally, under the microscopic observation, the cells dyed to blue were determined as dead, while the colourless and transparent ones were judged as live.

### 2.6 MTT test

Each hole of 96‐well culture plates was added with 100 μL cell suspension that contained 2 × 10^3^ cells, and the cells were cultured in 5% CO_2_ at 37°C. After each hole was added with 50 μg MTT, the cells were again cultured for another 4 hours. Then, the supernatants were sucked out before the addition of 200 μl DMSO. Ultimately, the optical densities (A values) of each hole at the wavelength of 570 nm were measured with utilization of microplate reader at the 24th, 48th, 72nd and 96th hours, respectively. The inhibition rate (IR) of cell proliferation was calculated according to the following formula:

$$\begin{array}{cc} \text{IR} & =[\left(A\;{\text{value}}_{\mathrm{testing}\;\mathrm{hole}}-A\;{\text{value}}_{\mathrm{blank}\;\mathrm{hole}}\right)\\ & \;/(A\;{\text{value}}_{\mathrm{contrast}\;\mathrm{hole}}-A\;{\text{value}}_{\mathrm{blank}\;\mathrm{hole}})]\times 100\%. \end{array}$$

### 2.7 Transwell test

Transwell chambers were placed within 24‐well plates, and the bottom membranes were coated with the diluted Matrigel. Serum‐free DEME nutrient solution was used to dilute cell sediments, and the concentration was adjusted to 5 × 10³/mL. The upper and lower Transwell chambers were, respectively, added with 200 μL cell suspension and 500 μL DEME solution that included 10% foetal bovine serum. After being cultured for another 12 hours, cells were washed with pre‐cooled PBS for twice. Methyl alcohol was prepared to immobilize membranes and cells for 15 minutes, which were then dyed with 1% crystal violet for 30 minutes. Under the high‐power microscope, the invasive cells below the chamber membrane were quantified, and 5 views were randomly observed to get the average results.

Besides, the migration experiment was conducted mostly in accordance with the above procedures, except that Matrigel glue was hardly applied.

### 2.8 Cell apoptosis

The cells at the logarithmic phase were seeded within the culture vessels at the density of 1.25 × 10^5^/mL. Flow cytometry was employed to detect the cell apoptotic conditions, following the instructions of Annexin V‐ fluorescein isothiocyanate (FITC)/propidium iodide (PI) kit. The resultant scatter diagram was explained in the following way: (1) the left‐lower quadrant indicated live cells marked with FITC‐/PI−; (2) the left‐upper quadrant indicated live cells marked with FITC‐/PI+; (3) the right‐upper quadrant indicated dead cells marked with FITC+/PI+; and (4) the right‐lower quadrant indicated the early apoptotic cells marked with FITC+/PI−.

### 2.9 Dual‐luciferase reporter gene assay

PCR was performed to amplify the miR‐7 fragment that embraced the binding site for ciRS‐7. Subsequently, the products were cloned into the pmirGLO Dual‐luciferase miRNA Target Expression Vector, deriving wild‐type miR‐7 (ie miR‐7‐wt). Besides, the same putative binding site was mutated, and miR‐7‐Mut was drawn. With assistance of Lipofectamine 2000 (Invitrogen, Foster city, CA, USA), the cells were, respectively, transfected with pcDNA‐ciRS‐7 and pcDNA‐ciRS‐7‐ir and pRL‐TK reporter gene carrier. As for the targeted relationship between miR‐7 and RELA, the RELA fragment that covered the binding site of miR‐7 was also amplified by PCR before being cloned into the pmirGLO Dual‐luciferase Expression Vector (Promega, MA, USA), in which way the reporter vector RELA‐wild‐type (ie RELA‐wt) was constructed. Similarly, the RELA with the mutated binding site of miR‐7 was called RELA‐mutated‐type (ie RELA‐Mut). Subsequently, the cells were, respectively, transfected with miR‐7 mimics and miR‐NC, and each group was then transfected with RELA‐Wt 3′UTR‐PGL3 plasmid/RELA‐Mut 3′UTR‐PGL3 plasmid and pRL‐TK reporter gene carrier, respectively. The activities of Firefly and Renilla luciferase were evaluated successively by applying dual‐luciferase reporter assay system (Promega) in 48 hours after transfection. All the assays were repeated for at least 3 times.

### 2.10 Western blotting

By means of the 10% SDS‐PAGE electrophoresis, the extracted total protein was transferred onto nitrocellulose membranes for 60 minutes. Then, PVDF membranes were sealed at room temperature for 2 hours, and rabbit anti‐human RELA primary antibody (1:1000; Cell Signalling Technology Corporation) was incubated overnight. Subsequently, the rat anti‐rabbit second antibody (1:5000; Abcam Corporation) was added and incubated for another 2 hours at room temperature. Then, enhanced chemiluminescence (ECL) was applied for exposure and development, and gel image analysis system (model number: GelDoc 2000; Bio‐Rad, USA) was employed to analyse the electrophoresis strips. The β‐actin was regarded as the control of ciRS‐7 regarding calculation of the intensity values. All the assays were repeated for at least 3 times.

### 2.11 Statistical analysis

All the statistical analyses were conducted with SPSS 20.0 software. The enumeration data were compared with χ^2^ test, while measurement data (mean ± SD) were contrasted using *t*‐test. The method of Kaplan‐Meier was applied to fit overall survival (OS) curves of the included subjects. It would be considered statistically significant when *P* value was <.05.

## 3 RESULTS

### 3.1 CiRS‐7 and miR‐7 expressions within NSCLC tissues and cell lines

**Figure 1:**
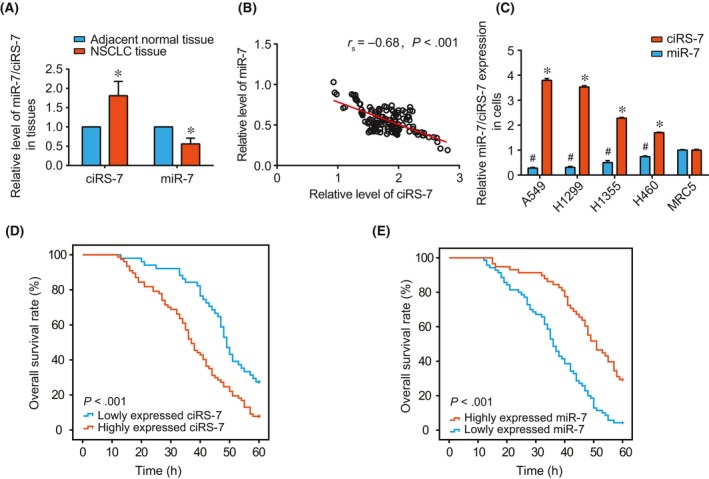
CiRS‐7 and miR‐7 expressions within non‐small cell lung cancer (NSCLC) tissues and cells. (A) CiRS‐7 and miR‐7 expressions were, respectively, compared between NSCLC tissues and adjacent normal tissues; *: *P* < .05. (B) CiRS‐7 expressions were correlated with miR‐7 expressions within NSCLC tissues. (C) CiRS‐7 and miR‐7 expressions were, respectively, compared between NSCLC cell lines (ie A549, H1299, H1355 and H460) and the normal human embryonic lung fibroblast cell line (ie MRC5); *: *P* < .05 when compared with ciRS‐7 expression within MRC5 cell line; #: *P* < .05 when compared with miR‐7 expression within MRC5 cell line. The correlation between ciRS‐7 (D) or miR‐7 (E) expressions and the over survival (OS) rates of patients with NSCLC

It was indicated in Figure 1A that the ciRS‐7 expression within NSCLC tissues rose far beyond that within para‐carcinoma tissues (*P* < .05), whereas the miR‐7 expression followed an inverse trend (*P* < .05). The different trends also rendered a significantly negative correlation between ciRS‐7 and miR‐7 expressions within NSCLC tissues (*P* < .05) (Figure 1B). Analogously, the within NSCLC cell lines (ie A549, H1299, H1355 and H460 cell lines) were associated with strikingly higher ciRS‐7 expressions and significantly lower miR‐7 expressions, when compared with the normal human embryonic lung fibroblast cell line (ie MRC5 cell line) (*P* < .05) (Figure 1C).

### 3.2 Association of ciRS‐7 and miR‐7 expressions with NSCLC patients’ OS

The incorporated patients with NSCLC were divided into higher ciRS‐7 expression (>average ciRS‐7 expression) group and lower ciRS‐7 expression (<average ciRS‐7 expression) group (Table 2). Simultaneously, they were also separated into higher miR‐7 expression group (>average miR‐7 expression) and lower miR‐7 expression (<average miR‐7 expression) group. It was derived that highly expressed ciRS‐7 and lowly expressed miR‐7 both exhibited significant correlations with advanced histopathological grade, larger tumour size and severer lymph node metastasis (*P* < .05). Moreover, Kaplan‐Meier analysis also manifested that NSCLC subjects with highly expressed ciRS‐7 or lowly expressed miR‐7 possessed inferior OS to ones with lowly expressed ciRS‐7 or highly expressed miR‐7 (*P* < .05) (Figure 1D,E). Furthermore, ciRS‐7, miR‐7, histopathological grade, lymph node metastasis and histopathological stage were probably the independent prognostic factors for NSCLC (all *P* < .05) (Table 3).

**Table 2:** The correlation between ciRS‐7/miR‐7 expressions and the NSCLC patients’ baseline clinical characteristics

Characteristics	ciRS‐7 expression			miR‐7 expression		
	Low	High	*P* value	Low	High	*P* value
N = 128	51	77		70	58	
Age						
≤60	26	31		34	23	
>60	25	46	.232	36	35	.312
Gender						
Female	17	28		26	19	
Male	34	49	.725	44	39	.605
Histopathological type						
Adenocarcinoma	28	30		29	29	
Squamous	23	47	.076	41	29	.332
Histopathological grade						
I + II	42	31		29	44	
III	9	46	**<.001**	41	14	**<.001**
Inflammation						
Yes	11	17		16	12	
No	40	60	.946	54	46	.768
Lymphovascular invasion						
Yes	17	21		20	18	
No	34	56	.463	50	40	.761
Tumour size						
T1	19	10		8	21	
T2‐4	32	67	**.001**	62	37	**<.001**
Lymph node metastases						
N0‐1	40	47		42	45	
N1‐2	11	30	**.039**	28	13	**.034**
Distant metastases						
M0	48	64		61	51	
M1	3	13	.065	9	7	.893
Histopathological stage						
I + II	36	38		32	42	
III + IV	15	39	**.017**	38	16	**.002**

NSCLC, non‐small cell lung cancer. The bold values indicate statistically significant results.

**Table 3:** The relationship between specific characteristics and the NSCLC patients’ overall survival

Characteristics	Univariate analysis			Multivariate analysis		
	Hazard ratio	95% CI	*P* value	Hazard ratio	95% CI	*P* value
ciRS‐7 expression						
Low vs High	**2.144**	**1.44‐3.20**	**<.001**	**1.705**	**1.02‐2.86**	**.043**
miR‐7 expression						
Low vs High	**0.355**	**0.24‐0.53**	**<.001**	**0.552**	**0.36‐0.85**	**.007**
Histopathological grade						
I + II vs III	**6.846**	**4.43‐10.57**	**<.001**	**2.571**	**1.43‐4.63**	**.002**
Tumour size						
T1 vs T2‐4	**3.781**	**2.21‐6.48**	**<.001**	1.71	0.94‐3.11	.079
Lymph node metastases						
N0‐1 vs N1‐2	**2.23**	**1.50‐3.32**	**<.001**	**1.66**	**1.07‐2.59**	**.025**
Histopathological stage						
I + II vs III + IV	**4.228**	**2.84‐6.29**	**<.001**	**3.987**	**2.47‐6.43**	**<.001**

NSCLC, non‐small cell lung cancer. The bold values indicate statistically significant results.

### 3.3 CiRS‐7 and miR‐7 regulated proliferation, invasion, migration and apoptosis of NSCLC cells

**Figure 2:**
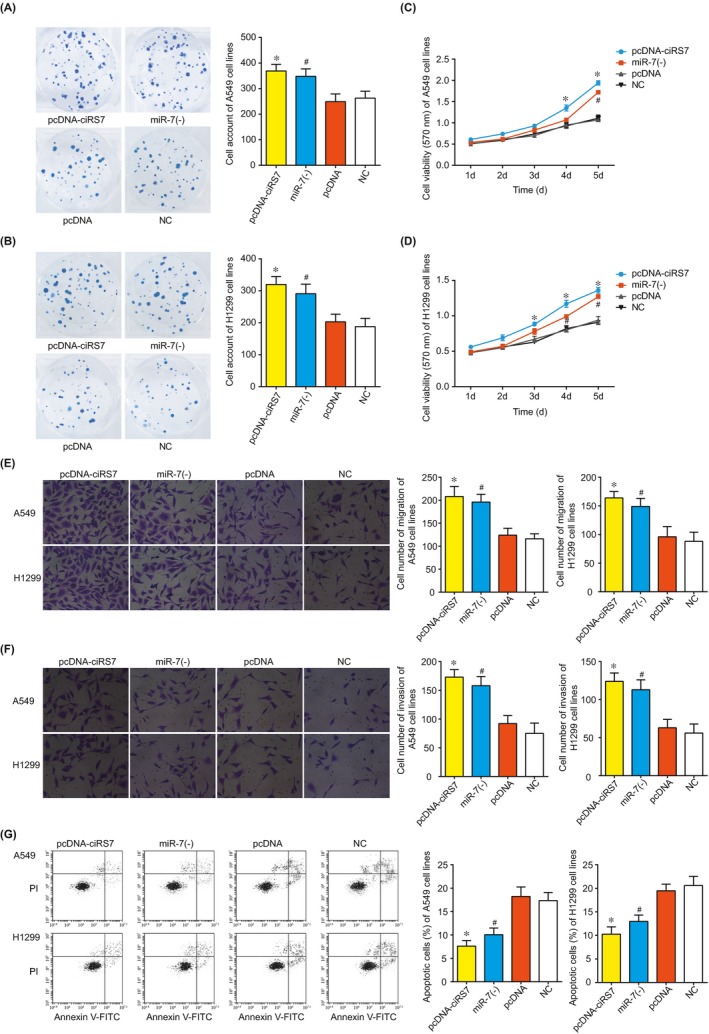
The viability of A549 and H1299 cell lines among groups of pcDNA‐ciRS‐7, miR‐7 (‐), pcDNA and NC according to the experimental results of trypan blue cell count (A‐B) and MTT (C‐D). The migratory (E), invasive (F) and apoptotic (G) statuses of A549 and H1299 cell lines among groups of pcDNA‐ciRS‐7, miR‐7 (‐), pcDNA and NC.*: *P* < .05 when compared with pcDNA; #: *P* < .05 when compared with NC

The cell number of pcDNA‐ciRS‐7 group escalated faster than that of pcDNA group, and miR‐7(‐) group displayed a better multiplicative capacity than NC group (*P* < .05) (Figure 2A,B). Meanwhile, the cell viability of pcDNA‐ciRS7 group and miR‐7(‐) group both exceeded that of pcDNA group and NC group (*P* < .05) (Figure 2C,D). In addition, pcDNA‐ciRS7 group and miR‐7(‐) group were associated with stronger invasive and migratory abilities of NSCLC cells than pcDNA group and NC group (*P* < .05) (Figure 2E,F). Correspondingly, the apoptotic rates of pcDNA‐ciRS7 group and miR‐7(‐) group were largely depressed in comparison with pcDNA group and NC group (*P* < .05) (Figure 2G).

### 3.4 The targeted relationship between ciRS‐7 and miR‐7

**Figure 3:**
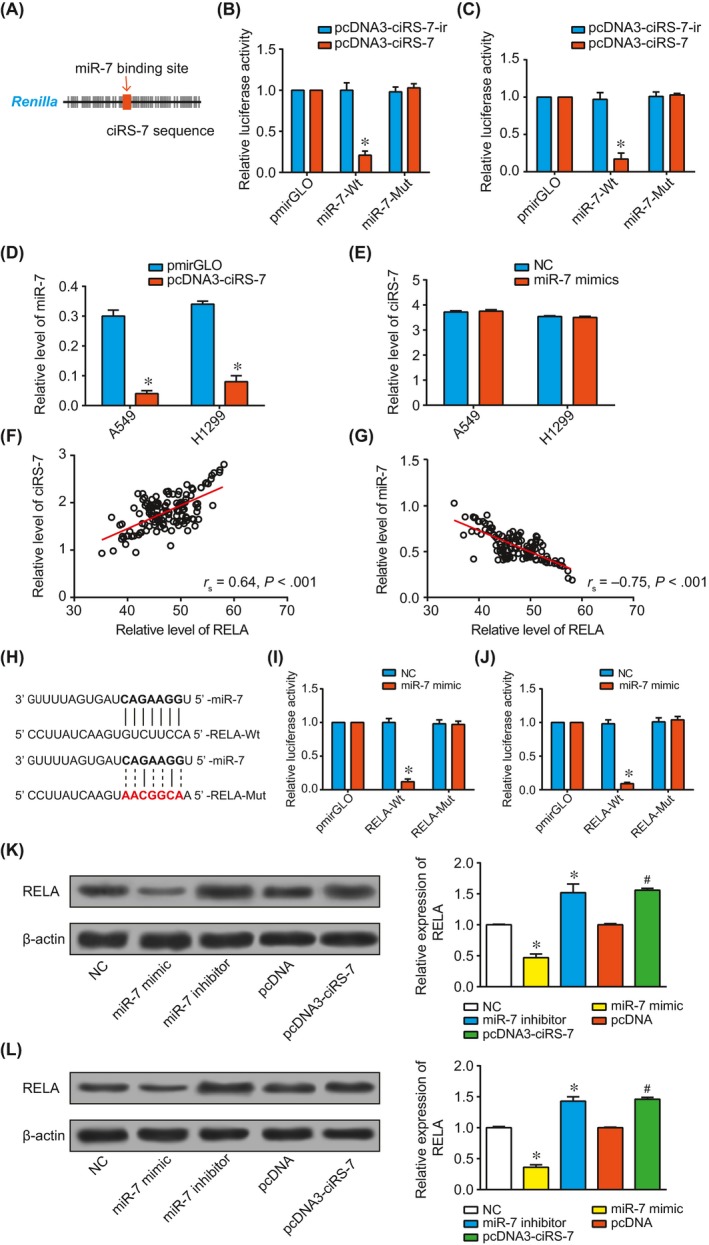
The relationship between ciRS‐7 and miR‐7. (A) CiRS‐7 was targeted by miR‐7 in the specific binding site. The relative luciferase activity of A549 (B) and H1299 (C) cell lines were detected after transfection with pcDNA3‐ciRS‐7 + miR‐7‐mut or pcDNA3‐ciRS‐7 + miR‐7.*: *P* < .05 when compared with NC. (D) The impacts of pcDNA3‐ciRS‐7 on miR‐7 expressions were evaluated within A549 and H1299 cell lines.*: *P* < .05 when compared with pmirGLO. (E) The impacts of miR‐7 mimic on ciRS‐7 expressions were evaluated within A549 and H1299 cell lines. The correlation between miR‐7 and RELA: (F) CiRS‐7 expression was positively correlated with RELA expression within non‐small cell lung cancer (NSCLC) tissues, (G) MiR‐7 expression was negatively correlated with RELA expression within NSCLC tissues. (H) MiR‐7 was targeted by RELA in the specific binding sites. The relative luciferase activity of A549 (I) and H1299 (J) cell lines were determined after transfection with miR‐7 mimic + RELA‐Mut or miR‐7 mimic + RELA‐Wt. The RELA expression was determined among groups of pcDNA3‐ciRS‐7, pcDNA, miR‐7 inhibitor, miR‐7 mimic and NC within A549 (K) and H1299 (L) cell lines. *: *P* < .05 when compared with NC; #: *P* < .05 when compared pcDNA

Based on the prediction results of Starbase 2.0 software (http://starbase.sysu.edu.cn/seedTargetInfo.php?type=circRNA&database=hg19&name=hsa-miR-7-5p&geneName=CDR1-AS_hsa-circRNA8162&autoId=94962&orgTable=mirCircRNAInteractionsAll), altogether 45 targeted sites were shared by ciRS‐7 and miR‐7 (Figure 3A). The A549 and H1299 cells that were cotransfected with pcDNA3‐ciRS‐7 and miR‐7‐wt were accompanied by significantly decreased luciferase activity than ones transfected with pcDNA3‐ciRS‐ir (*P* < .05), yet the cells cotransfected with pcDNA3‐ciRS‐7 and miR‐7‐mut hardly generated this change (Figure 3B,C). Moreover, up‐regulated ciRS‐7 expressions within A549 and H1299 cells could markedly cut down the miR‐7 expression (*P* < .05), yet down‐regulated miR‐7 expression could barely modify the expression level of ciRS‐7 (Figure 3D,E).

### 3.5 MiR‐7 targeted NF‐KB subunit (ie RELA) to modify its expression

**Figure 4:**
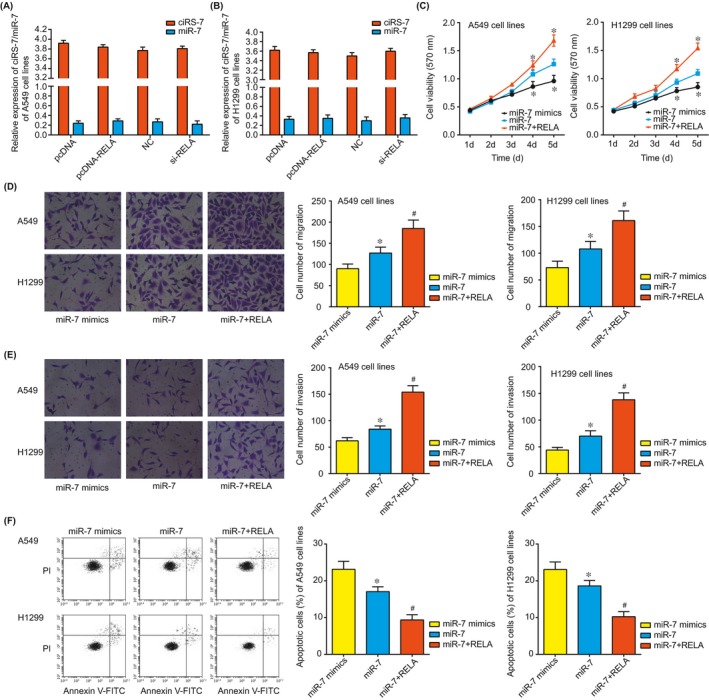
The effect of pcDNA‐RELA or si‐RELA on ciRS‐7 and miR‐7 expressions within A549 (A) and H1299 (B) cell lines, as well as the role of miR‐7 mimic, miR‐7 and miR‐7 + RELA in regulating the viability of A549 (C) and H1299 (D) cell lines.*: *P* < .05 when compared with miR‐7 group. The migratory (D), invasive (E) and apoptotic (F) conditions of A549 and H1299 cell lines among groups of miR‐7 mimic, miR‐7 and miR‐7 + RELA. *: *P* < .05 when compared with miR‐7 mimic; #: *P* < .05 when compared with miR‐7

As Figure 3H–J was indicated, overexpression of miR‐7 contributed to attenuated luciferase activity of RELA3′‐UTR reporter constructs, but this phenomenon could not be observed when the RELA3′‐UTR was mutated. More than that, miR‐7 expression within NSCLC tissues presented negative correlations with RELA expressions (*P* < .05), while ciRS‐7 expression went up with the rising of RELA expression (*P* < .05) (Figure 3F,G). Concerning the in‐vitro experiments, the aberrantly uplifted ciRS‐7 expression and lowered miR‐7 expression could elevate RELA expression significantly (Figure 3K,L); nonetheless, modulating RELA expression imposed little effects on ciRS‐7 and miR‐7 expressions (Figure 4A,B). In short, it was implied that RELA was subject to regulation of ciRS‐7 and miR‐7 within NSCLC cells.

### 3.6 MiR‐7 modulated proliferation, invasion, migration and apoptosis of NSCLC cells through functioning on NF‐KB subunit (ie RELA)

Interestingly, the optical density (OD) value of miR‐7 + RELA group was dramatically higher than that of miR‐7 group (*P* < .05), suggesting that RELA suppressed the lowered effect of miR‐7 on NSCLC cells’ activity (Figure 4C). Following a similar trend, miR‐7 + RELA group was correlated with more desirable migratory and invasive abilities of NSCLC cells than miR‐7 group (*P* < .05) (Figure 4D,E). Finally, the NSCLC cells’ apoptosis rate of miR‐7 + RELA group was significantly lower than that of miR‐7 group (*P* < .05) (Figure 4F).

## 4 DISCUSSION

It was generally accepted as neoplastic invasion and migration when tumour cells aggressively grew from primary lesions to surrounding tissues, blood circulation and lymphatic circulation.^25^ Interestingly, ncRNAs (eg circRNA, lncRNA and miRNA) have been documented to regulate cancers’ progression through modulating DNA structures, transcription of RNAs and translation of proteins.^26^, ^27^

As a matter of fact, competitive endogenous RNAs (ceRNAs), which mainly incorporated lncRNAs and circRNAs, were revealed to compete with miRNA for the miRNA recognition elements (MREs) of target mRNAs, so as to reduce the control over target genes and to affect the development of diseases.^24^, ^28^, ^29^, ^30^ Distinct from miRNA, circRNA was impregnable to RNA exonuclease, as its special circular‐closure structure made itself more stable than linear RNA.^31^, ^32^, ^33^, ^34^, ^35^ Hence, circRNAs were more likely to be implicated within the mechanisms that gave rise to dysfunctional bioactivities, such as the onset and aggravation of cancers. For instance, ciRS‐7, the natural antisense transcript of cerebellar degeneration‐associated protein 1 (CRD1), contained approximately 70 MREs of miR‐7.^24^, ^32^, ^36^ Its high expression within cytoplasms could effectively lower miR‐7 activity through binding to it, thereby down‐regulating the expressions of insulin‐like growth factor‐1 receptor (IGF1R), epidermal growth factor receptor (EGFR) and focal adhesion kinase (FAK).^37^, ^38^, ^39^

It was also documented that miR‐7, which was positioned on chromosome 15, held up the growth of lung cancer cells (ie A549) through down‐regulating Bcl‐2 expression.^40^ Webster et al^41^ also speculated that miR‐7 might directly restrain the expression of v‐raf‐1 murine leukemia viral oncogene homologue and the Raf‐related signal transmission, finally controlling the proliferation of lung cancer cells. Furthermore, it was miR‐7 that hindered the migration of lung cancer through inhibiting phosphatidylinositol‐3‐kinase (PI3K) and Toll‐like receptor 9 (TLR9) expressions.^16^ Based on the above‐mentioned researches, it could be summarized that ciRS‐7 combined with miR‐7 might modify proliferation, invasion and migration of cancer cells, although hardly any studies have associated ciRS‐7 with miR‐7 within NSCLC‐relevant investigations.

Correspondingly, ciRS‐7 and miR‐7 expressions were also detected within NSCLC tissues and cells here, validating that they could be widely and stably expressed within biological cells. Also, the apparent differences of ciRS‐7 and miR‐7 expressions between NSCLC and adjacent normal tissues suggested that they might play a role in the occurrence and development of NSCLC. In addition, the negative association of ciRS‐7 with miR‐7 further provided hints that ciRS‐7 might function as the suppressor of miR‐7 or ceRNA that constrained miR‐7 expression. Furthermore, relevant experiments of trypan blue staining cell counting, MTT, Transwell and flow cytometry indicated that ciRS‐7 promoted migration, invasion and proliferation of NSCLC cells, and inhibited apoptosis of the cells. Nonetheless, the role of miR‐7 was the opposite to ciRS‐7 regarding the above end indicators.

Moreover, the Western blot experiments in this study (Figure 3K,L) demonstrated that RELA (ie NF‐kB‐p65) was the downstream molecule of ciRS‐7 and miR‐7 within NSCLC cells. In addition, it was rational to believe that ciRS‐7 and miR‐7 acted on NF‐kB signalling to regulate the invasion, migration, proliferation and apoptosis of NSCLC cells, as the activation of NF‐kB could promote proliferation of tumour cells and adherence of the cells to vascular cells via facilitating expressions of the molecules relevant to antiapoptosis and proadhesion.^42^ For example, NF‐kB appeared to be involved in the process of tumour cells’ proliferation, differentiation and apoptosis through up‐regulating the expressions of matrix metalloproteinases (MMPs) to degrade extracellular matrix (ECM).^43^

All in all, the current investigation logically linked ciRS‐7 with miR‐7 and NF‐kB signalling in accelerating proliferation, invasion and migration of NSCLC cells, yet several shortcomings were still evident. Firstly, recruiting a larger population of different ethnicities could make the study result be generalized to a wider range of crowds. Secondly, additional molecules, besides NF‐kB‐p65, should be explored to validate the role of NF‐kB signalling in modulating NSCLC development. Finally, animal models should further be established to confirm the study result, so that ciRS‐7 might become crucially diagnostic and treatment biomarkers for NSCLC.

## CONFLICT OF INTEREST

The authors declare that no conflict interest existed.
